# Initial Diagnosis and Management for Acute Charcot Neuroarthropathy

**Published:** 2018-11-29

**Authors:** Matthew L. Vopat, Michelle J. Nentwig, Alexander C.M. Chong, Jamie L. Agan, Naomi N. Shields, Shang-You Yang

**Affiliations:** 1 University of Kansas School of Medicine-Wichita, Department of Orthopaedics, Wichita, KS; 2 Via Christi Health, Wichita, KS; 3 Advanced Orthopaedics Associates, Wichita, KS

**Keywords:** Charcot, neuroarthropathy, diabetic foot, diabetic neuropathy

## INTRODUCTION

Charcot neuroarthropathy, also known as Charcot foot, is a complication of diabetes mellitus where there is progressive degeneration of the joints, but it potentially is devastating in its consequences.^1^ It commonly affects the middle of the foot, hind-foot joints, the ankle, and forefoot joints, and it is believed to result from inflammation in the foot that becomes abnormally protracted due to the underlying neuropathy.^2^–^8^ The prevalence of Charcot neuroarthropathy is up to 13% in individuals with diabetes.^9^–^11^ Patients with Charcot neuroarthropathy encounter increased morbidity and decreased quality of life and mortality.^2^,^4^,^5^,^12^,^13^ If there is a delay in treatment, Charcot neuroarthropathy could result in ulceration and infection which can lead to amputation of the limb.^12^–^16^ These patients have a significant financial impact on the health care system through primary care, community care, outpatient costs, increased bed occupancy, and prolonged stays in hospital.

Charcot neuroarthropathy poses many clinical challenges in its diagnosis and management. The often asymptomatic nature of the condition is very similar to ankle sprain, cellulitis, venous thrombosis, inflammatory arthritis, or gout in a healthy patient.^5^,^16^–^22^ Missed diagnosis is as high as 79% which ultimately leads to a delay in treatment for an average of 29 weeks.^11^,^16^,^17^,^20^,^23^–^25^

Charcot neuroarthropathy is caused by multiple factors, but essentially it is the result of peripheral neuropathy which is a complication associated with many diseases.^2^,^4^,^5^ The underlying peripheral neuropathy can skew the pain perception the patient experiences and can mislead the clinician on their differential diagnosis of an “inflamed foot”. A thorough neurological examination of the foot can uncover the underlying inflammatory and osteolytic disease process of Charcot neuroarthropathy.^2^,^4^,^11^,^19^,^26^–^29^

Early recognition and intervention is imperative to avoid the rapid progression toward permanent foot deformity, ulceration, and the possibility of limb loss.^16^,^30^,^31^ There are multiple review articles about Charcot neuroarthropathy^2^,^11^–^13^,^16^,^23^,^25^,^28^,^32^–^34^, but a lack of guidance on foot screen strategies for primary care and emergency room physicians. There is a need for a comprehensive guideline for initial diagnoses and management on foot care to advocate for increased awareness, thereby leading to earlier diagnosis and treatment by a multi-disciplinary team.

In the current study, a thorough literature review of Charcot neuroarthropathy was conducted to evaluate efficacious methods of protocol design and potential barriers to implementation. The literature review also encompassed treatment goals for patients with Charcot neuroarthropathy. Based on the literature review, a foot screen strategies protocol for Charcot neuroarthropathy was devised by the authors and reported here. This protocol contains three parts: (1) pathophysiology of acute Charcot neuroarthropathy to highlight the relationship between the clinical findings and the development of the disease, (2) a comprehensive guideline on how to screen and evaluate Charcot neuroarthropathy, and (3) a brief overview on prevention of Charcot neuroarthropathy in patients with diabetes and other forms of peripheral neuropathy.

### Pathophysiology

The underlying cause for Charcot neuroarthropathy is due to peripheral neuropathy, which is a loss of function of the nerves in the periphery of the body.^2^–^4^ The primary episode of inflammation can result from a number of factors, but ultimately leads to an increase in pro-inflammatory cytokines (interleukin-1β and tumor necrosis factor-α) which leads to receptor activator of the nuclear factor-κB ligand (RANKL-NFκB) pathway. Osteoclasts are activated leading to bone lysis followed by clearing of debris. In the presence of autonomic neuropathy, there is increased blood flow to the area, which acts to clear away bony material demineralizing the bone, cartilage, and soft tissue in the region.^3^ However, in the presence of diabetic neuropathy, the patient does not have the protective pain perception. Therefore, they continue to walk on the inflamed foot exacerbating the progressive pathway of osteolysis and osteopenia and weakening the pedal skeleton, leading to the high risk for dislocation and/or fracture.^5^–^8^,^34^,^35^

### Charcot neuroarthropathy screening guideline

**Figure 1:**
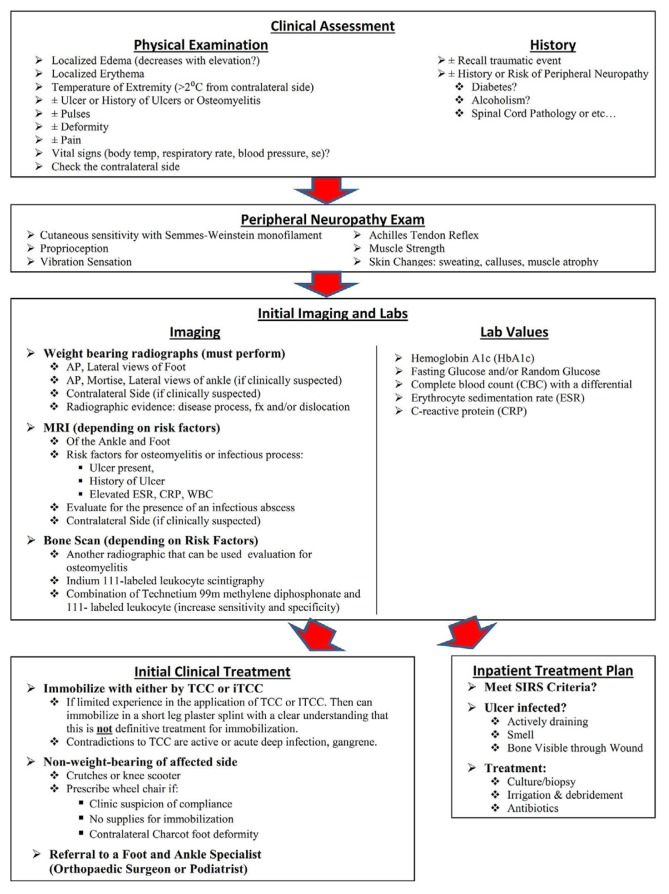
Charcot neuroarthropathy screening and management guideline.

Figure 1 shows the step-by-step process from initial diagnosis of a patient presenting with symptoms of an inflamed foot in a primary care setting to managing the patient with acute Charcot neuroarthropathy. The detailed pathway/algorithm for initial clinical diagnoses and management of acute Charcot neuroarthropathy should be divided into several phases: clinical assessment, peripheral neuropathy evaluation, initial imaging and lab studies, diagnosis, management, and recommendation. Each phase includes the how, the why, and a step-by-step guideline to making an early diagnosis easier and providing appropriate and immediate management for these patients.

### Clinical assessment

A high degree of suspicion of Charcot neuroarthropathy is necessary with thorough history and physical examination when a patient presents with an acute erythematous, warm, or edematous foot, with or without any significant history of trauma or surgery, especially for patients with diabetes and peripheral neuropathy with these symptoms.^36^

### History

A thorough patient history of a traumatic event or peripheral neuropathy should be assessed. Approximately 50% of patients with Charcot neuroarthropathy would remember a precipitating, minor traumatic event, and if no traumatic episode was recalled, the time frame for which the patient noticed changes in their foot shape and/or gait should be documented. About 25% of patients develop similar changes in the contralateral foot.^11^,^17^,^18^,^22^,^30^,^34^,^37^–^39^ Often, the precipitating factor for acute Charcot neuroarthropathy is not a traumatic event, but rather repetitive micro-trauma on an insensate foot.^11^,^17^,^18^,^29^

A chronic history of diabetes longer than 10 years has a strong association with peripheral neuropathy and potential development of Charcot neuropathy.^11^,^12^,^34^,^40^–^44^ Due to the strong association between elevated hemoglobin A1c (HbA1c) and the development of Charcot neuropathy, the patients’ compliance to their diabetic treatment should be assessed and documented.^2^ Some patients also may be unaware of an underlying diagnosis of diabetes at the time of presentation, thereby diabetes screening is essential. Other potential causes of peripheral neuropathy also should be evaluated such as alcohol abuse, syringomyelia, spinal pathology, vitamin B12 deficiency, heavy metal poisoning, leprosy, tertiary syphilis, and idiopathic form.^2^,^3^,^8^,^45^ Other potential risk factors that can lead to the development of Charcot neuroarthropathy include obesity, advanced age, renal failure, iron deficiency, osteoporosis, and rheumatoid arthritis.^2^,^3^,^8^,^45^

### Physical examination

The classical physical examination findings for an acute Charcot neuroarthropathy are often unilateral localized inflammatory symptoms of the foot, such as edema, erythema, and increased foot temperature of the extremity.^2^–^4^,^11^,^21^,^22^,^28^,^34^,^41^ A simple physical exam that can be helpful to distinguish between an infectious process and Charcot neuroarthropathy is to have the patient lay supine and elevate the affected extremity for 5 – 10 minutes. Localized edema will decrease with elevation of the extremity in Charcot neuroarthropathy while an infectious process is less likely to decrease.^11^,^46^,^47^

The infrared cutaneous temperature monitor to detect foot skin temperature changes is one of the most accurate tools for diagnosis acute Charcot neuroarthropathy. It may be used in the areas of forefoot, mid-foot, and hind-foot. A temperature difference of 2°C from the contralateral foot indicates an active Charcot neuroarthropathy.^48^–^50^

The presence of ulcers or a history of ulcers indicates the need to screen for an active infection. Signs and symptoms, such as purulence, foul smell, or wet gangrene, should be noted.^2^,^4^,^51^ An ulcer with the size over 2 cm2 and visualization of bone increases the risk of developing and/or presence of osteomyelitis.^52^,^53^

Clinical assessments such as foot tenderness, pedal pulses, and foot deformity should be evaluated.^2^–^4^,^11^,^21^,^22^,^28^,^34^,^41^ Cutaneous changes such as increased sweating, calluses, and muscle atrophy should be documented.^2^ Owing to the possible presence of peripheral neuropathy, pain may not always be present; with only 50% of patients reporting pain.^28^,^36^,^37^

Charcot neuroarthropathy can present as an infectious process and screening of the patient’s vital signs for systemic signs of infection such as fever, chills, elevated heart rate or respiratory rate can be helpful.^2^,^4^,^54^ However, lack of these symptoms may not rule out an infectious process.

### Peripheral neuropathy examination

**Figure 2:**
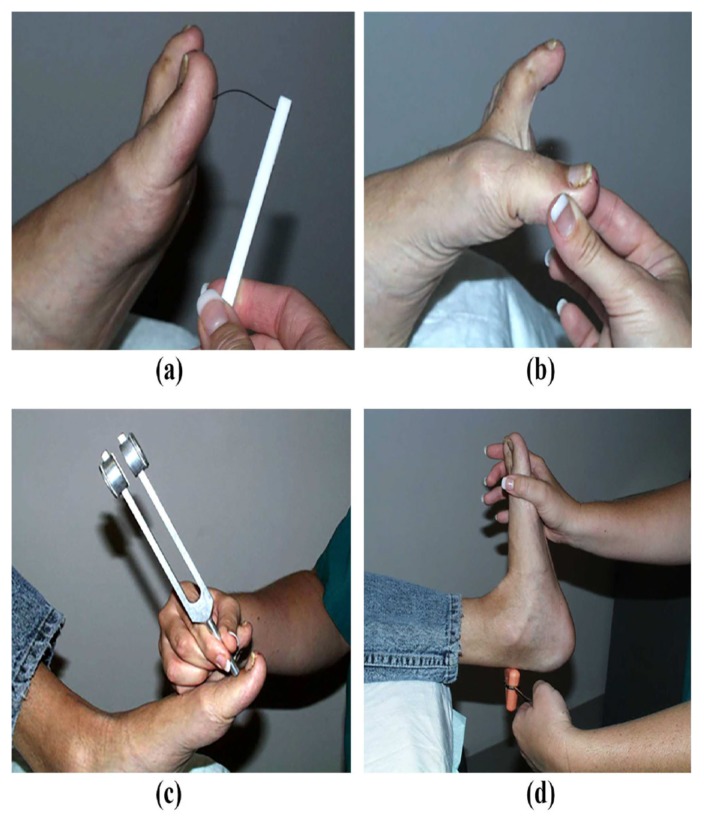
Neurologic examinations. (a) Semmes-Weinstein Monofilament test; (b) proprioception test on a big toe; (c) tuning fork vibration sensation test; and (d) Achilles reflex test using a reflex hammer.

The existence of little or no pain may mislead the patient and physician^38^, as peripheral neuropathy is likely to be an essential prerequisite for the onset of the Charcot neuroarthropathy process. Bilateral neurologic examination should be assessed for numbness, paresthesia, and dysesthesia by evaluating cutaneous sensitivity using Semmes-Weinstein monofilament, proprioception, tuning fork vibration sensation, or Achilles tendon reflex (Figure 2).^2^,^4^,^55^–^57^ The Semmes-Weinstein monofilament test is a noninvasive, low-cost, rapid, and easy-to-apply test that is the most sensitive test in diagnosing peripheral neuropathy.^55^,^56^ The locations for this test on both feet include the first, third, and fifth metatarsal heads and plantar surface of the distal hallux and third toe, but avoid callused areas. Neuropathy usually starts in the first and third toes and progresses to the first and third metatarsal heads. Seven or less of 10 different touch sensation locations on the patient’s foot is an indication of peripheral neuropathy.^52^,^56^,^58^

### Imaging

**Figure 3:**
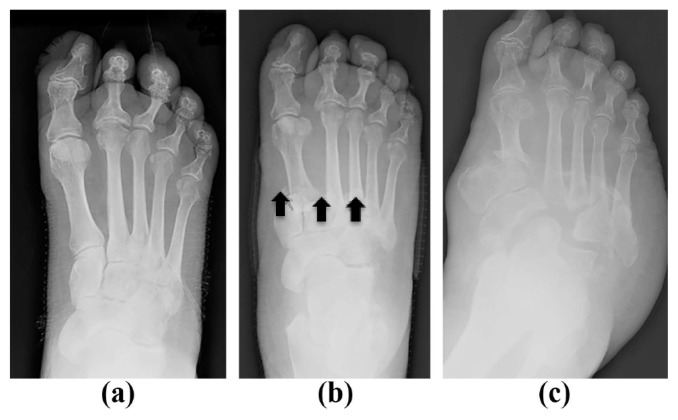
Progression of a foot Charcot neuroarthropathy on plain radiographs: (a) Initial anteroposterior view of an acute Charcot neuroarthropathy foot; (b) 6-month follow-up, which shows the persistent and progressive joint effusion, narrowing of the joint space, soft tissue calcification, minimal subluxation, osteopenia, and bone fragmentation; and (c) 2-year follow-up, which shows severe destruction of the foot without proper management.

Radiographs are the primary imaging method for initial evaluation of the foot in patients, as they provide information on bone structure, alignment, and mineralization.^4^,^15^,^59^,^60^ They also are useful in diagnosing the pathology, locating the area of involvement, evaluating quality of bone, and identifying if the process is acute or chronic. It is essential to get plain radiographs on patients present with a symptomatic foot. Unfortunately, radiographic changes of Charcot neuroarthropathy typically are delayed and have low sensitivity. The plain radiographs can be negative for up to three weeks with the only finding being soft tissue swelling. Figure 3 shows an example of the Charcot neuroarthropathy progression on plain radiographs.

The initial radiographic images should include anteroposterior and lateral weight-bearing views of the affected foot and/or full series ankle views (anteroposterior, mortise, and lateral views) depending on clinical suspicion.^4^,^60^,^61^ Evidence of demineralization, bone destruction, and periosteal reaction on plain radiographic images can lead towards a diagnosis of Charcot neuroarthropathy, although this also can be seen in chronic osteomyelitis.

If Charcot neuroarthropathy is suspected, magnetic resonance imaging (MRI) allows detection of subtle changes in the early stages when the plain radiographic images appears normal.^25^ MRI also is useful to rule out osteomyelitis, especially in the presence of an ulcer, history of ulcers, elevated erythrocyte sedimentation rate (ESR), C-reactive protein (CRP), or leukocytosis.^2^,^5^,^25^,^45^,^62^–^69^ The sensitivity and specificity are reported greater than 77% and 80% respectively in differentiating acute Charcot neuroathropathy from osteomyelitis.^2^,^45^,^62^–^68^ Osteomyelitis on MRI often displays diffuse marrow involvement that usually only affects a single bone like the metatarsal heads and the calcaneus,^4^,^70^ whereas Charcot neuroarthropathy more classically exhibits periarticular and subchondral bone marrow edema affecting several joints.^4^,^71^

Bone scan is another imaging tool that can be used to differentiate osteomyelitis from Charcot neuroarthropathy. A technetium-99m methylene diphosphonate scintigraphy is less useful than leukocyte scintigraphy because there is enrichment on both osteomyelitis and Charcot neuroarthropathy, whereas leukocyte scintigraphy is only positive in osteomyelitis.^2^,^62^ The combination of technetium-99m methylene diphosphonate scintigraphy with indium-111 white blood cells, labeled leukocyte scintigraphy may improve sensitivity (87%) and specificity (81%) for differentiating acute Charcot neuroarthropathy and osteomyelitis.^4^,^59^,^72^–^75^

### Laboratory tests

There is a strong association between the duration of diabetes, elevated HbA1c, and the development of Charcot neuroarthropathy.^9^,^11^,^76^ The patient should be screened initially for uncontrolled diabetes by evaluating fasting glucose, HbA1c, and/or random glucose levels. Even if the patient has no known diabetes history, they should be screened because of the high prevalence of diabetes.^25^,^33^,^77^ If these lab values are not elevated and the patient has no known diabetes, then further evaluation should be made for the cause of peripheral neuropathy.

Initial lab orders should include complete blood count (CBC) with a differential, erythrocyte sedimentation rate (ESR), and C-reactive protein (CRP). Elevations in ESR, CRP, and leukocytosis are more in line with an infectious process like osteomyelitis.^2^,^3^,^34^,^78^ An ESR greater than 70 mm/h has an 11-fold increased risk for the presence of osteomyelitis.^52^,^79^ A slight elevation in ESR with normal white blood cell count (WBC) may occur in Charcot neuroarthropathy.^80^ Normal inflammatory markers may be noticed occasionally in chronic osteomyelitis; the diagnosis may depend on other modalities like radiographs and MRI.^2^,^51^

### Treatment

The most important aspect of the success for a foot screen protocol is early management of these suspected Charcot neuroarthropathy patients. Many cases of acute Charcot neuroarthropathy are mistreated because the condition is not recognized widely outside specialist clinics. If the suspected Charcot neuroarthropathy is complicated by ulceration or infection, then an inpatient treatment plan should be implemented before sending the patient home. The gold standard of conservative management strategy for Charcot neuroarthropathy has been immobilization and non-weight bearing.^81^

### Inpatient treatment plan

Patients with confirmed or suspected infection, such as cellulitis, deep tissue infection, abscess or osteomyelitis, should be admitted for evaluation, when they have at least two of the following criteria from Systemic Inflammatory Response Syndrome (SIRS): body temperature ≥ 38°C or < 36°C, heart rate > 90 beats/minute, respiratory rate > 20 breaths/min. or arterial carbon dioxide tension (PaCO2) < 32 mmHg, abnormal white blood cell count ≥ 12,000/μL or ≤ 4,000/μL or > 10% immature (bands) forms.^82^–^84^ Infection in the Charcot neuroarthropathy patient poses great challenges. Discussion with a foot and ankle specialist is recommended about treatment plans such as irrigation and debridement, culture/biopsy of the wound, and antibiotic treatment. Immobilization of the affected foot continues until complete resolution of the acute phase. Patient education regarding the diagnosis, estimated length of treatment, and expected outcomes is an important component of Charcot neuroarthropathy management. If the patient understands the nature of this limb-threatening condition, they may be more motivated to adhere to the management plan. Emphasis on the importance of strict immobilization and attending regular follow-up reviews may improve the outcome of Charcot neuroarthropathy.

### Initial clinical treatment

**Figure 4:**
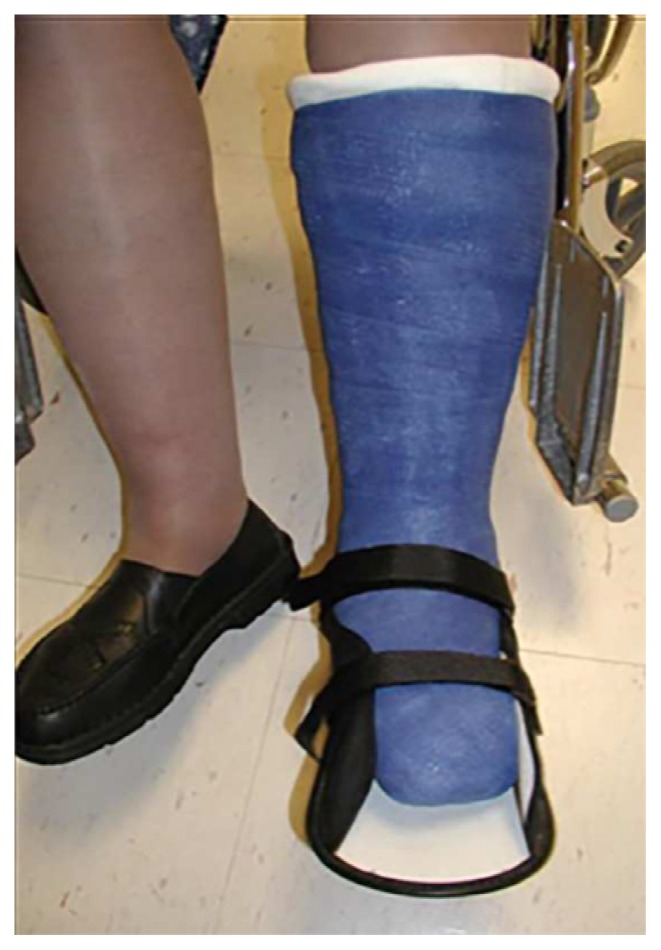
Total contact cast (TCC).

The initial clinical treatment for a patient suspected of Charcot neuroarthropathy should be immobilization and non-weight bearing of affected foot.^2^,^4^,^11^,^15^,^81^,^85^–^87^ The goals are to stop the inflammation-mediated damage, relieve pain, and maintain or protect the skeleton of the foot and ankle from further deformity on the affected limb until definitive diagnosis can be made. The use of a total contact cast (TCC; Figure 4), instant total contact cast (iTCC) with the use of crutches, or a knee scooter is recommended. If the clinician has limited experience in the application of TCC or iTCC, they can immobilize the patient in a short leg splint with a clear understanding that this is not the definitive treatment for immobilization. A wheelchair should be prescribed in cases where there is clinical suspicion of non-compliance or a question of bilateral involvement. These treatments are not definitive and the patient should be referred to a foot and ankle specialist (orthopedic or podiatrist) to establish a multidisciplinary team approach for definitive treatment.

### Preventive medicine

Patients with diabetes mellitus and mild-to-severe peripheral neuropathy have high potential of developing Charcot neuroarthropathy. It affects 415 million people globally. This number is predicted to rise to 642 million by 2040.^88^ These patients have a significant impact on health care costs, so prevention is important.^11^,^25^,^76^,^89^ Like most complications of diabetes, the key is to control patients’ glucose and HbA1c levels either by diet and/or medication.^90^ The American Diabetes Association 2016 guidelines^90^ recommended a glycemic target of HbA1c < 7.0% (53 mmol/mol), preprandial capillary plasma glucose of 80 – 130 mg/dL (4.4 – 7.2 mmol/L), and peak postprandial capillary plasma glucose < 180 mg/dL (10 mmol/L) for non-pregnant adults.

Patient education is an essential component of the long-term management, focusing on the importance of appropriate footwear, offloading, regular follow up reviews, and the risk of further complications.^27^,^28^ Lifestyle changes for obesity, nutrition, smoking and alcohol abuse should be addressed.^2^ A thorough diabetic foot exam to check for any skin abnormalities and a neurological exam should be performed at least two times a year, if the patient shows signs of peripheral neuropathy. It is recommended that the patient be prescribed a hard shoe, diabetic foot wear, or foot orthoses, and advised against wearing sandals to prevent development of Charcot neuroarthropathy. Footwear is an important component of the long-term management of the insensate chronic Charcot neuroarthropathy, ensuring that it remains accommodated and protected. Patients should be educated on regular self-examinations of their feet for skin break down, swelling, erythema and ulcers, and encouraged to evaluate their shoes for any foreign bodies before putting them on.

## CONCLUSION

A thorough neurological examination must be a part of the physical exam for any patient presenting with unilateral erythema, edema, and increased foot temperature that has high risk factors for peripheral neuropathy. This examination could prevent any hidden inflammatory process, like Charcot neuroarthropathy, from going undiagnosed. A protocol for primary care and emergency room physicians provides a comprehensive guideline on foot screening, especially for acute Charcot neuroarthropathy.
